# Panretinal Congenital Hypertrophy of the RPE in an 8-Year-Old Girl with an X-Linked STAG2 Mutation

**DOI:** 10.3390/jcm14176110

**Published:** 2025-08-29

**Authors:** Maximilian D. Kong, Mohamed M. Sylla, Jin Kyun Oh, Vaidehi S. Dedania, Megan Soucy, Aykut Demirkol, Scott E. Brodie, Irene H. Maumenee, Stephen H. Tsang

**Affiliations:** 1 Edward S. Harkness Eye Institute, Department of Ophthalmology, Columbia University Irving Medical Center, New York-Presbyterian Hospital, New York, NY 10032, USA; maximilian.kong@downstate.edu (M.D.K.); mohamed.sylla@downstate.edu (M.M.S.);; 2 Jonas Children’s Vision Care, and Bernard & Shirlee Brown Glaucoma Laboratory, Department of Ophthalmology, Columbia University, New York, NY 10032, USA; 3 College of Medicine, SUNY Downstate Health Sciences University, Brooklyn, NY 11203, USA; 4 Department of Ophthalmology, NYU Grossman School of Medicine, New York, NY 10016, USA; 5 Edward S. Harkness Clinical Coordinating Center, Columbia University, New York, NY 10032, USA

**Keywords:** congenital hypertrophy of the retinal pigment epithelium (CHRPE), stromal antigen 2 (STAG2), inherited retinal disease (IRD), optical coherence tomography (OCT), autofluorescence, electroretinogram (ERG)

## Abstract

**Introduction**: Congenital hypertrophy of the retinal pigment epithelium (CHRPE) is a benign proliferation of the melanin-producing retinal pigment epithelium (RPE). Although often a benign and incidental finding, multifocal CHRPE may mimic lesions associated with familial adenomatous polyposis (FAP). **Case Description**: We describe an 8-year-old girl presenting with optic disc pallor and widespread multifocal bear track CHRPE observed bilaterally on dilated fundoscopy. Fundus autofluorescence (FAF) imaging showed uniform areas of hypoautofluorescence corresponding to the bear track lesions. Spectral domain optical coherence tomography (SD-OCT) demonstrated normal lamination without atrophy. The full-field electroretinogram (ffERG) was within normal limits. Whole-genome sequencing (WGS) revealed a likely pathogenic heterozygous variant in the *STAG2* gene (c.3222dup, p.Ser1075IlefsTer12). **Conclusions**: We present a rare case of bilateral, panretinal bear track CHRPE in a child with a likely pathogenic variant in *STAG2*. Using multimodal imaging, we contrast bear track lesions of the retina with FAP-associated CHRPE. We also present possible ophthalmic manifestations in carriers of pathogenic *STAG2* variants.

## 1. Introduction

Congenital hypertrophy of the retinal pigment epithelium (CHRPE) is a benign proliferation of melanin-producing retinal pigment epithelium (RPE) cells [1,2]. Overall, the prevalence in the general population is estimated to be 1.2% [3]. Typically, CHRPE presents as a unifocal, flat, and hyperpigmented retinal lesion often discovered incidentally on fundus examination. Multifocal CHRPE is a less common manifestation that is characterized by clustering of smaller CHRPE lesions (100–300 μm) from the disc to the periphery, creating the characteristic appearance of bear tracks [2,4]. Although often benign, multifocal CHRPE should warrant additional investigation due to systemic associations with familial adenomatous polyposis (FAP) and Gardner syndrome, where it manifests in up to 90% of cases [5,6]. Given the potential risk of future malignancy, differentiating between benign bear tracks and atypical, FAP-associated CHRPE is an important diagnostic consideration.

The present case also introduces a possible novel genetic association. The *STAG2* gene encodes a subunit of the cohesin complex, which is involved in the cohesion of sister chromatids during cell division, DNA repair, and modulation of gene expression [7,8]. Loss-of-function variants in cohesin genes can result in multisystem developmental disorders known as cohesinopathies, which most likely arise due to the cohesin complex’s role in gene expression during embryogenesis. Specifically, loss-of-function in *STAG2* is associated with X-linked Mullegama–Klein–Martinez syndrome (MIM# 301043) and X-linked holoprosencephaly (MIM# 301022), with significant phenotypic variability across males and females [9]. *STAG2* is highly expressed in the prosencephalic neural folds of the developing embryo, a tissue that later gives rise to the RPE among other neural tissues. Genes involved in early neural differentiation may influence not only the structure and development of the brain, but also that of the RPE and retina. The ophthalmic manifestations of disease are not well characterized. Here, we report a rare case of panretinal bear track CHRPE in an 8-year-old girl with a likely pathogenic frameshift variant in *STAG2*, using multimodal imaging to distinguish these lesions from FAP-associated CHRPE.

## 2. Methods

Patient evaluation included a thorough medical and family history, as well as a comprehensive ophthalmic examination with measurement of best corrected visual acuity (BCVA), intraocular pressure, anterior segment evaluation, and dilated fundus examination following pharmacologic dilation with phenylephrine (2.5%) and tropicamide (1%). Multimodal imaging included color fundus photography, fundus autofluorescence (FAF), and spectral domain optical coherence tomography (SD-OCT). Digital color fundus photographs were obtained (Zeiss Clarus 700, Carl Zeiss Meditec, Jena, Germany). FAF was acquired using Zeiss Clarus 700 (Carl Zeiss Meditec, Jena, Germany). SD-OCT was acquired using a Spectralis HRA + OCT (Heidelberg Engineering, Heidelberg, Germany). Full-field electroretinogram (ffERG) was performed in-house using Dawson, Trick, and Litzkow (DTL) electrodes and Ganzfeld stimulation on a Diagnosys Espion Electrophysiology System (Diagnosys LLC, Littleton, MA, USA) according to international standards [10]. ffERG photopic responses were categorized as within normal limits, attenuated, or extinguished based on a clinician’s interpretation of a detectable waveform on the 30 Hz ERG, with reproducible phases; scotopic responses were based on a detectable waveform on the 0.01 dark-adapted ERG [11,12]. Proband-only whole-genome sequencing (WGS) was performed using peripheral blood through the New York Genome Center. Interpretation of variants followed ACMG guidelines [13]. Variant information was queried using the gnomAD v3.1.2 database to assess allele frequency [14]. In silico prediction of variant pathogenicity was performed using combined annotation-dependent depletion (CADD v1.7) [15]. Informed consent was waived due to the minimal risk conferred to the patient and the retrospective nature of the study design as per the Institutional Review Board at Columbia University (protocol AAAB6560). All procedures were reviewed and in accordance with the tenets of the Declaration of Helsinki.

## 3. Background

An 8-year-old girl was referred to Columbia University Irving Medical Center for evaluation of unusually extensive bear track lesions identified on routine eye exam. Her medical history was significant for difficulty swallowing as an infant and speech delay, which improved with speech-language therapy. Family history revealed Sephardic Jewish ancestry and a paternal uncle with known colonic polyps but no bear track lesions. Both parents had undergone unremarkable screening colonoscopies. There was a further history of two miscarriages of unknown sex. One of the patient’s four older sisters had a history of developmental delay, including poor feeding in infancy and speech delay requiring therapy.

## 4. Clinical Findings

**Figure 1:**
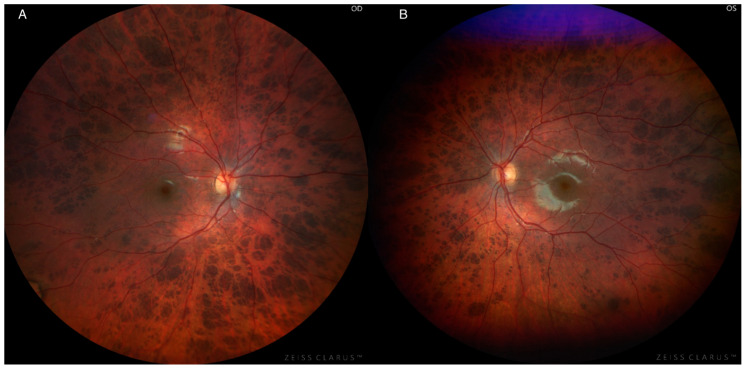
Color fundus photography of an 8-year-old girl with panretinal, bilateral bear tracks, showing temporal pallor and round, flat, hyperpigmented lesions spanning the fundus, including the macula. Lesions start small closer to the disc and then increase in density and size in the periphery. OD = right eye (**A**); OS = left eye (**B**).

On examination, best corrected visual acuity (BCVA) was 20/20 in both eyes. Intraocular pressure (IOP) was measured as 18 mmHg in the right eye and 20 mmHg in the left eye. Dilated fundus examination found temporal optic nerve pallor and diffuse bear tracks extending into the macula with an increase in density and size of the lesions in the periphery (Figure 1A,B).

## 5. Investigations

**Figure 2:**
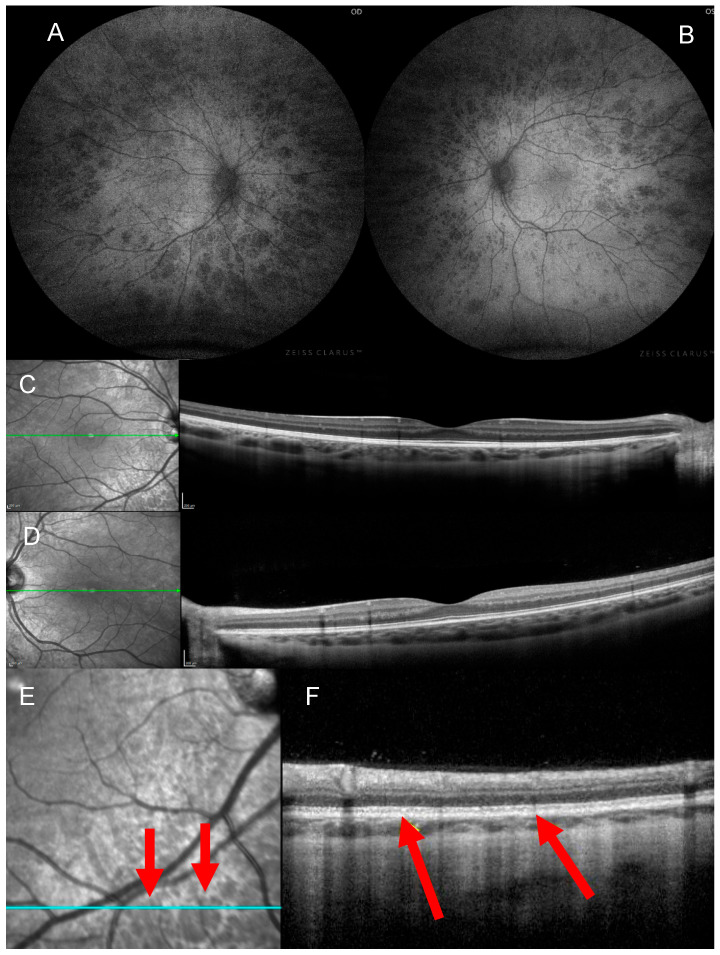
Autofluorescence and optical coherence tomography imaging of an 8-year-old girl with panretinal, bilateral bear tracks. Autofluorescence imaging of the right (**A**) and left eye (**B**) revealed small areas of hypoautofluorescence, consistent with the hyperpigmented lesions observed on fundoscopy. Spectral domain optical coherence tomography imaging (**C**,**D**) revealed organized lamination with no photoreceptor layer atrophy, retinal pigment epithelium thickening, or hyperreflectivity at the level of the lesions denoted by red arrows (**E**,**F**).

**Figure 3:**
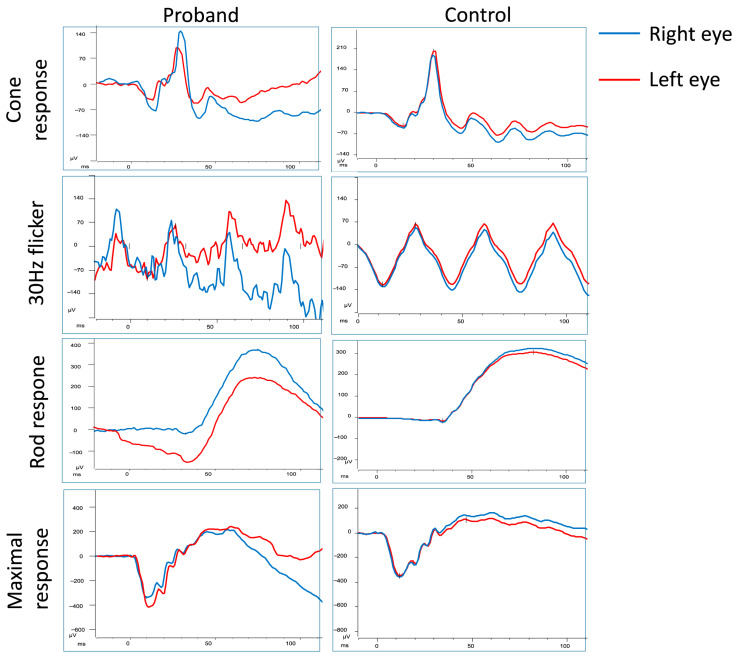
Full-field electroretinogram (ffERG) recordings of an 8-year-old girl with panretinal, bilateral bear track retina. ffERG revealed normal rod and cone amplitudes with no implicit time delay.

Autofluorescence imaging showed uniformly hypoautofluorescent spots corresponding with the bear track lesions (Figure 2A,B). Spectral domain optical coherence tomography (SD-OCT) demonstrated otherwise normal retinal architecture (Figure 2C,D). A ffERG was performed on-site and showed normal scotopic and photopic responses (Figure 3). Proband-only whole-genome sequencing (WGS) revealed a likely pathogenic heterozygous variant in the *STAG2* gene (c.3222dup, p.Ser1075IlefsTer12). The patient’s clinical findings are summarized in Table 1.

## 6. Outcome and Follow-Up

At 2-year follow-up, the patient’s vision, exam findings, and imaging all remained stable. Retinal examination and imaging of the patient’s sisters were unremarkable.

## 7. Discussion

We present a rare case of bilateral bear track CHRPE, a pattern that can mimic FAP-associated CHRPE, but is typically benign. Additionally, we draw a potentially novel retinal association with female carriers of pathogenic *STAG2* mutations. FAP-associated CHRPE presents as multiple, bilateral lesions haphazardly distributed throughout the retina. The lesions are nonuniform in pigmentation and are oval or pisciform, with a fishtail-shaped area of hypopigmentation. In contrast, our patient exhibited uniformly pigmented, well-demarcated lesions, consistent with benign bear tracks, despite their uncommon bilateral distribution [22]. On autofluorescence, both FAP-associated and bear track CHRPE appear hypoautofluorescent. However, FAP-associated lesions may have a hyperautofluorescent halo or tail, whereas bear tracks often appear uniformly hypoautofluorescent [23]. OCT findings of FAP-associated CHRPE often reveal RPE extension into the retina, hyperreflectivity and thickening of the RPE, and atrophy of the overlying photoreceptor layer at the level of the lesion [24]. Choroidal elevation, excavations, and serous detachments at the level of the lesions have also been reported [25]. In contrast, RPE cells in bear tracks are normal-sized, and OCT shows normal lamination with no photoreceptor layer atrophy (Figure 2F) [22,24].

Once FAP-associated CHRPE has been excluded, management of bear tracks is conservative, as the lesions are asymptomatic and carry a very low risk of malignancy. Patients with bear tracks do not have an increased risk of developing colon cancer compared to the general population [26]. The restricted surface area of the lesions in bear track CHRPE also explains the overall normal rod and cone function seen in the patient’s ffERG (Figure 3) [27]. However, the development of scotomas as a result of RPE neovascularization has rarely been reported [22,28]. Follow-up visits to monitor for lesion progression, and infrequent visual field testing for prospective new visual field defects are recommended [29].

Pathogenic variants in *STAG2* are associated with X-linked Mullegama–Klein–Martinez syndrome and X-linked holoprosencephaly [7,16]. Mullegama–Klein–Martinez syndrome manifests with developmental delay, microcephaly, and congenital anomalies. A summary of genotype–phenotype correlations is outlined in Table 1. Pathogenic variants in *STAG2* are generally lethal or result in severe disease in males, while females can manifest with a wide spectrum of severity, likely due to variable patterns of X-inactivation [7,9]. A previous case series by Kumar et al. found that female carriers ranged from mild intellectual disability, including speech delay, to normal cognitive function [9]. The majority of this cohort demonstrated normal growth and development. Other features reported included short stature, small head circumference, and facial hypotonia. Our patient’s history of difficulty swallowing and speech delay aligns with this spectrum. Further, the absence of male siblings and the presence of two miscarriages may reflect male lethality associated with dysfunction in *STAG2*. Interestingly, the patient’s sisters did not have any retinal findings on exam, although one required therapy for poor feeding as an infant and speech therapy as a toddler.

While some studies in *STAG2*-related disease have reported ocular abnormalities such as strabismus, there is a paucity of detailed retinal phenotyping [7,16,17,18,19,20,21]. One prior report described optic nerve hypoplasia, which may account for the temporal nerve pallor observed in this patient [7]. Additionally, Freyberger et al. reported an atrophic retinal scar in a male patient with a pathogenic *STAG2* variant [20]. Unfortunately, neither study included retinal imaging for comparison.

The multisystem congenital abnormalities observed in cohesinopathies primarily arise from impaired gene expression during embryogenesis, as mutations causing overt failure of chromosomal segregation or DNA repair are likely to be lethal [8,30]. The most common cohesinopathy, Cornelia de Lange syndrome, is caused by dysfunction in a variety of cohesin subunits, such as NIPBL, SMC1A, SMC3, RAD21, BRD4, HDAC8, and ANKRD11 [31]. Indeed, human cell lines and murine models have shown greatly altered gene expression during development, while chromatid cohesion and DNA repair remained largely intact [32]. As part of Cornelia de Lange syndrome, patients may have ocular manifestations such as ptosis, strabismus, high myopia, and nystagmus [30,31].

Functional studies in other cohesinopathies, including *STAG2*-related disease, have shown similar support for altered gene expression as the primary cause of syndromic abnormalities rather than poor chromatid cohesion [7,16]. Using Giemsa staining, Mullegama et al. found that while some mutant cells with a loss-of-function *STAG2* variant actually displayed tighter chromatid cohesion, premature separation did not occur [16]. Furthermore, Kruszka et al. showed that the cohesin genes *STAG2* and *SMC1A* are highly expressed in the prosencephalic neural folds during primary neurulation in mouse embryos, supporting their role in forebrain development [7]. This tissue gives rise to the retina, RPE, and optic nerve, suggesting a potential developmental link with ocular dysgenesis. Supporting this, Gibellato et al. reported ocular manifestations from dysfunction in *SMC1A*, with visual impairment observed in 25% of a large international cohort of *SMC1A* epilepsy syndrome [33].

This patient’s variant is absent from population databases (gnomAD v3.1.2), suggesting it is not a common benign variant in the general population [14]. The c.3222dup variant in *STAG2*, located in exon 30 of this 35-exon gene, is predicted to result in a frameshift with a premature stop codon (p.Ser1075IlefsTer12) leading to loss-of-function via nonsense-mediated decay. In silico prediction with CADD (v1.7, GRCh38) yielded a score of 34 for the patient’s variant, supporting a high likelihood of pathogenicity [15]. Additionally, although most reported loss-of-function variants in the literature are upstream of c.3222, a nearby downstream loss-of-function variant has been identified in an affected individual [19]. Future in vitro and in vivo studies must be performed to properly characterize this variant’s effects.

This report is limited by the single-subject design and the absence of functional validation of the reported *STAG2* variant. While the c.3222dup variant is predicted to result in loss-of-function via nonsense-mediated decay, supporting experimental data is lacking. Future development of mouse models or human cell lines is vital for the validation of this novel variant’s pathogenicity. Giemsa staining of patient-derived cells would also be useful in determining levels of chromatid cohesion. Additional neuroimaging for both the patient and family members would have been useful in supporting our findings. Further genetic testing of family members, including verification of the Y chromosome in miscarried fetuses, could provide stronger support for the X-linked inheritance pattern and associated male lethality.

## 8. Conclusions

This case of panretinal CHRPE in the context of a likely pathogenic *STAG2* mutation highlights a rare retinal presentation that mimics FAP-associated CHRPE and expands upon the phenotypic spectrum of *STAG2*-associated disease. It also highlights the utility of ophthalmic examination and WGS in diagnosing patients with genetic diseases that may carry potential systemic manifestations. Multimodal imaging was essential in distinguishing benign bear track CHRPE from syndromic forms, supporting conservative management. Future studies should explore the prevalence of retinal pigmentary abnormalities, including CHRPE, in individuals with *STAG2* variants to assess for potential phenotypic patterns. Functional assays and animal models could also help clarify whether *STAG2* expression directly contributes to retinal development and RPE morphology.

## Figures and Tables

**Table 1:** Summary of reported STAG2 variants with associated systemic and ocular findings. Data are compiled from the present study and previously published case reports and series describing genotype–phenotype correlations, with ophthalmologic features included when available. Abbreviations: PFO = patent foramen ovale; PDA = patent ductus arteriosus; VSD = ventricular septal defect; GERD = gastroesophageal reflux; CDH = congenital diaphragmatic hernia; NR = not reported.

	Reported Variant	Sex	Summary of Systemic Findings	Ocular Findings
Present Study	c.3222dup, p.Ser1075IlefsTer12	F	Feeding problems, speech delay	Optic nerve pallor, congenital hypertrophy of the RPE
Mullegama et al., 2017 [16]	c.205C > T, p.Arg69Ter	F	Dysgenesis of splenium and corpus callosum, speech delay, cleft palate, bilateral microtia, hearing loss, hemivertebrae, butterfly vertebrae, fifth finger clinodactyly, VSD	Strabismus
Kruszka et al., 2019 [7]	c.3034C > T, p.Arg1012Ter	F	Alobar brain, microcephaly, midline cleft palate, low-set ears, lumbar spina bifida, GERD	NR
	c.205C > T, p.Arg69Ter	F	Semi-lobar brain, global developmental delay, growth delay, microcephaly, cleft palate, micrognathia, PFO, PDA	NR
	c.436C > T, p.Arg146Ter	F	Alobar brain, growth delay, microcephaly, absent nose, hypognathia, hypoplastic ear, hemivertebrae, VSD, duodenal atresia	Cyclopia
	c.2533 + 1G > A (intronic)	F	Semi-lobar brain, microcephaly, hypoplastic left heart, double outlet right ventricle	NR
	c.2898_2899del, p.Glu968SerfsTer15	F	Microform brain, developmental delay, growth delay, microcephaly	NR
	c.775C > T, p.Arg259Ter	F	Septo-optic dysplasia, intellectual disability, motor delay, left hip dysplasia, VSD	Bilateral optic nerve hypoplasia
Yuan et al., 2019 [17]	c.418C > T, p.Gln140Ter	F	Seizure disorder, motor and speech delay, dysmorphic ears, vertebral clefts, hypoplastic left heart	NR
	c.1605T > A, p.Cys535Ter	F	Intellectual disability, motor and speech delay, growth delay, microcephaly, micrognathia, microtia, hearing loss, fifth finger clinodactyly	Strabismus
	c.1811G > A, p.Arg604Gln	F	Intellectual disability, growth delay, microcephaly, micrognathia, hypotonia, vertebral clefts, CDH, pulmonary hypoplasia, GERD	NR
	c.1658_1660delinsT, p.Lys533IlefsTer6	F	Microform brain with agenesis of the corpus callosum, seizure disorder, intellectual disability, motor and speech delay, growth delay, microcephaly with colpocephaly, single central incisor, micrognathia, dysmorphic ears, vertebral anomalies	NR
	c.476A > G, p.Tyr159Cys	M	Ectopic posterior pituitary, intellectual disability, growth delay, cleft palate, low-set ears, hypotonia, scoliosis, single kidney	None
Aoi et al., 2020 [18]	c.3097C > T, p.Arg1033Ter	M	Holoprosencephaly, cleft palate, hypoplastic left heart	NR
	c.2229C > T, p.Trp743Ter	F	White matter hypoplasia, seizure disorder, intellectual disability, growth delay, cleft palate, hearing loss, thoracic hemivertebrae	NR
Rinaldi et al., 2020 [19]	c.3724C > T, p.Arg1242Ter	F	Holoprosencephaly, low-set ears, CDH, ambiguous genitalia	Unspecified eye abnormalities
Freyberger et al., 2021 [20]	c.475T > C, p.Tyr159His	M	Polymicrogyria with hypotrophic corpus callosum, dolichocephaly, high arched palate, hypotonia	Atrophic retinal and uveal scar
Schmidt et al., 2022 [21]	c. 2184G > T, p.Gln728His	F	Hypotrophic right hemisphere with dysplastic corpus callosum and white matter hypoplasia, microcephaly, low-set ears, hearing loss, hemivertebrae, butterfly vertebrae, hemangioma	Limbal dermoid
	c.1412_1416 + 9del (intronic)	F	Holoprosencephaly, speech and motor delay, microcephaly, low-set ears, retrognathia, dystonia, scoliosis	Strabismus

## Data Availability

The datasets generated during and/or analyzed during the current study are available from the corresponding author on reasonable request.
